# Preoperative assessment of perforating arteries around amygdala glioblastoma using intra-arterial CT angiography with ultra-high-resolution CT and MR tractography: a case report

**DOI:** 10.1007/s00701-025-06741-y

**Published:** 2025-12-11

**Authors:** Gaku Inoue, Masayuki Kanamori, Shin-Ichiro Osawa, Yoshiteru Shimoda, Kazuki Shimada, Shingo Kayano, Yoshinari Osada, Shota Yamashita, Shunji Mugikura, Hidenori Endo

**Affiliations:** 1 Department of Neurosurgery, Tohoku University Graduate School of Medicine, 1‑1 Seiryo‑Machi, Aoba‑Ku, Sendai, Miyagi Japan; 2 Department of Radiological Technology, Tohoku University Hospital, Tohoku University, Sendai, Miyagi Japan; 3 Department of Diagnostic Radiology, Tohoku University Graduate School of Medicine, Sendai, Japan; 4 Department of Image Statistics, Tohoku Medical Megabank Organization, Tohoku University, Sendai, Japan

**Keywords:** Glioblastoma, Intra-arterial-CT angiography, Ultrahigh resolution CT; anterior choroidal artery

## Abstract

Preservation of the perforating arteries (PAs) supplying the pyramidal tract (PT-PAs) is essential during glioblastoma resection. A 60-year-old man presented with sensory aphasia caused by a left medial temporal glioblastoma. To determine the surgical indication and strategy, we assessed the tumor’s location and its relationship with the PT-PAs using intra-arterial computed tomography (CT) angiography combined with ultra-high-resolution CT (UHR-IA-CTA), gadolinium-enhanced T1-weighted magnetic resonance imaging, and tractography. Imaging revealed an amygdala glioblastoma without the involvement of PT-PAs. Total tumor resection was achieved without neurological deficits. This case highlights how integrating UHR-IA-CTA can guide surgical planning and enable safe, complete glioblastoma resection.

**Supplementary Information:**

The online version contains supplementary material available at 10.1007/s00701-025-06741-y.

## Introduction

Glioma resection in the medial temporal region encompassing the amygdala, hippocampus, and parahippocampal gyrus [2] remains challenging due to the proximity of critical neuronal and vascular structures [6, 13, 16, 17]. Tumors in this area may extend into the basal ganglia (BG) and involve perforating arteries (PAs) arising from the anterior choroidal artery (AchoA) and lenticulostriate arteries (LSAs) [13, 14]. Given that portions of LSA and the AchoA-derived PAs supply the pyramidal tract (PT), accurate assessment of the tumor’s relationship with these vessels is essential for determining surgical indications and planning. However, visualizing PT-supplying LSAs and AchoA perforators (PT-LSAs and PT-AchoAs, respectively) remains technically difficult because of their small diameter [9, 16]. Previous studies have demonstrated LSA and AchoA visualization using high-resolution computed tomography (CT) and high-field magnetic resonance imaging (MRI) [1, 5, 11, 12], but the precise anatomical course of PT-LSAs and PT-AchoAs has not been fully delineated owing to limited spatial resolution.

Recently, ultra-high-resolution CT (UHR-CT) with a maximum spatial resolution of 150 μm has been developed [7]. Using this technology, we established a novel CT angiography (CTA) technique—intra-arterial CTA with UHR-CT (UHR-IA-CTA)—combining intra-arterial contrast injection with UHR-CT to enhance visualization of LSAs, long insular arteries, and long medullary arteries. This approach enables direct identification of PT-LSAs by integrating fusion images with tractography [10].

In this report, we present a case of left amygdala glioblastoma in which the feasibility of maximal safe resection was determined by assessing the tumor’s relationship to the PT-LSAs and PT-AchoAs using fusion images derived from UHR-IA-CTA, gadolinium-enhanced T1-weighted MR imaging (Gd-T1WI), and diffusion tensor imaging (DTI).

## Case report

**Fig. 1:**
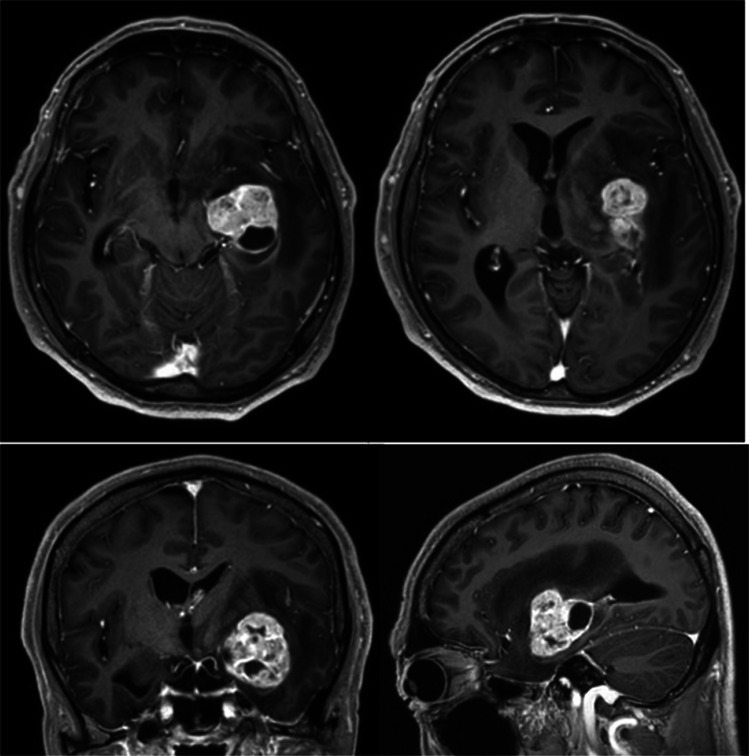
Preoperative axial (upper panel), coronal (lower left panel), and sagittal (lower right) gadolinium-enhanced T1-weighted magnetic resonance images showing a heterogeneously enhancing lesion in the left medial temporal lobe adjacent to the basal ganglia (BG). The exact boundary of the tumor and its invasion into the BG remain unclear

A 60-year-old man presented with progressive speech difficulty for over 3 months and was referred to our institution for evaluating a left medial temporal mass. On admission, the patient exhibited sensory aphasia without paresis. Gd-T1WI revealed a heterogeneously enhancing lesion in the left medial temporal region extending to the BG. Systemic contrast-enhanced CT revealed no evidence of other malignancies. A preoperative diagnosis of glioblastoma was made (Fig. 1).

The indication for maximal safe resection remained uncertain because it was unclear whether the tumor originated from or encased the PT-LSAs and PT-AchoAs. To clarify this, the anatomical relationship between the enhanced lesion and these vessels was assessed using UHR-IA-CTA, as previously described [10]. In brief, digital subtraction angiography (DSA) of the left cervical internal carotid artery (ICA) was performed to estimate capillary filling time and time-to-peak contrast. UHR-IA-CTA was then conducted using a UHR-CT scanner (Aquilion Precision; Canon Medical Systems, Otawara, Japan) with 9 mL of contrast medium injected over 3 s at 3 mL/s, followed by a 2 s scan delay. The obtained images were fused with 3D-Gd-T1WI and DTI data using Ziostation 2 (Ziosoft, Tokyo, Japan) and BrainLab Elements (BrainLab, Munich, Germany). The PT was reconstructed using DTI-based tractography. To enhance visualization, the LSAs, AchoA, their perforating arteries, the BG, internal capsule, and medial temporal region were manually segmented on the UHR-IA-CTA and fused with the 3D-Gd-T1WI and DTI data, eliminating cortical branch interference. The PT-LSAs and PT-AchoAs were identified on sequential 2-mm slab fusion images between UHR-IA-CTA and tractography, as well as on 3D maximal intensity projection (3D-MIP) vessel images overlaid with PT fiber tracts (3D-MIP-PT) in Ziostation 2. To visualize their spatial relationships, 3D reconstructions combining arteries from UHR-IA-CTA, the tumor from Gd-T1WI, and the PT from DTI tractography were created using BrainLab Elements.

**Fig. 2:**
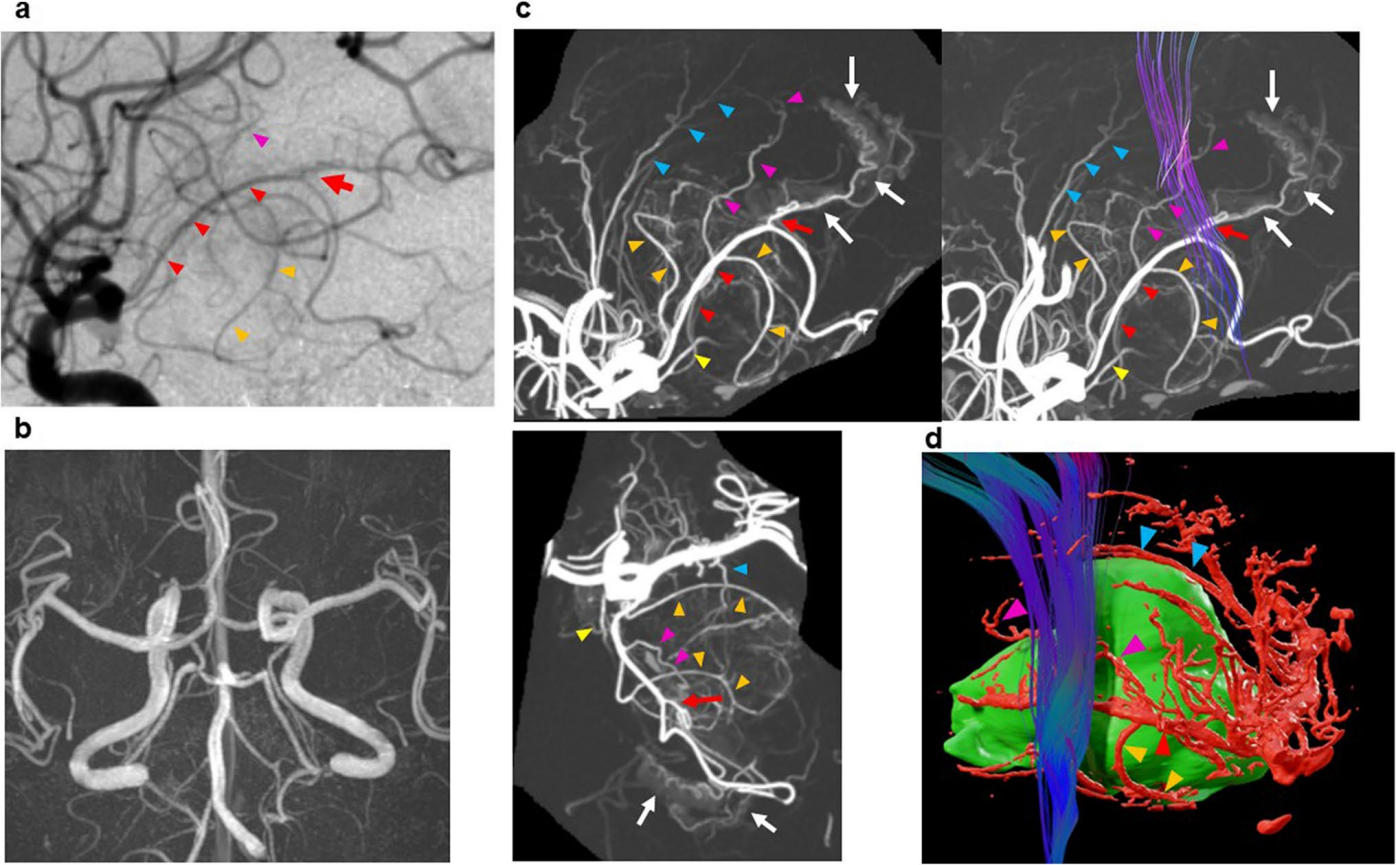
Vascular structures surrounding the tumor. **a** Digital subtraction angiography of the left internal carotid artery showing tumor staining from feeding arteries (orange arrowheads) arising from the anterior choroidal artery (AchoA, red arrowheads). The tumor was located anterior to the inferior choroidal point (red arrow). **b** Magnetic resonance (MR) angiography demonstrating a hypoplastic left posterior communicating artery (PcomA), with the posterior cerebral artery receiving blood flow through a well-developed left P1 segment. **c** Sagittal (upper left panel) and axial (lower panel) maximum intensity projection images from intra-arterial computed tomography (CT) angiography with ultra-high-resolution CT (UHR-IA-CTA). The pyramidal tract (PT) is visualized using diffusion tensor tractography (upper right panel). The lenticulostriate artery (LSA) and perforating arteries of the AchoA supplying the PT (PT-LSA and PT-AchoA, respectively) are clearly depicted (blue and pink arrowheads). Red, orange, and yellow arrowheads and red and white arrows indicate the AchoA, feeding arteries of tumor, PcomA, and inferior choroidal point, and choroid plexus, respectively. **d** Medial 3D reconstruction fusing UHR-IA-CTA arteries, gadolinium-enhanced T1-weighted MR tumor images, and PT tractography demonstrates the spatial relationship among the tumor, PT-LSA (blue arrowheads), PT-AchoA (pink arrowheads), feeding arteries (orange arrowheads), and AchA (red arrowhead). The PT-LSA and PT-AchoA were displaced anteriorly and medially, respectively

**Fig. 3:**
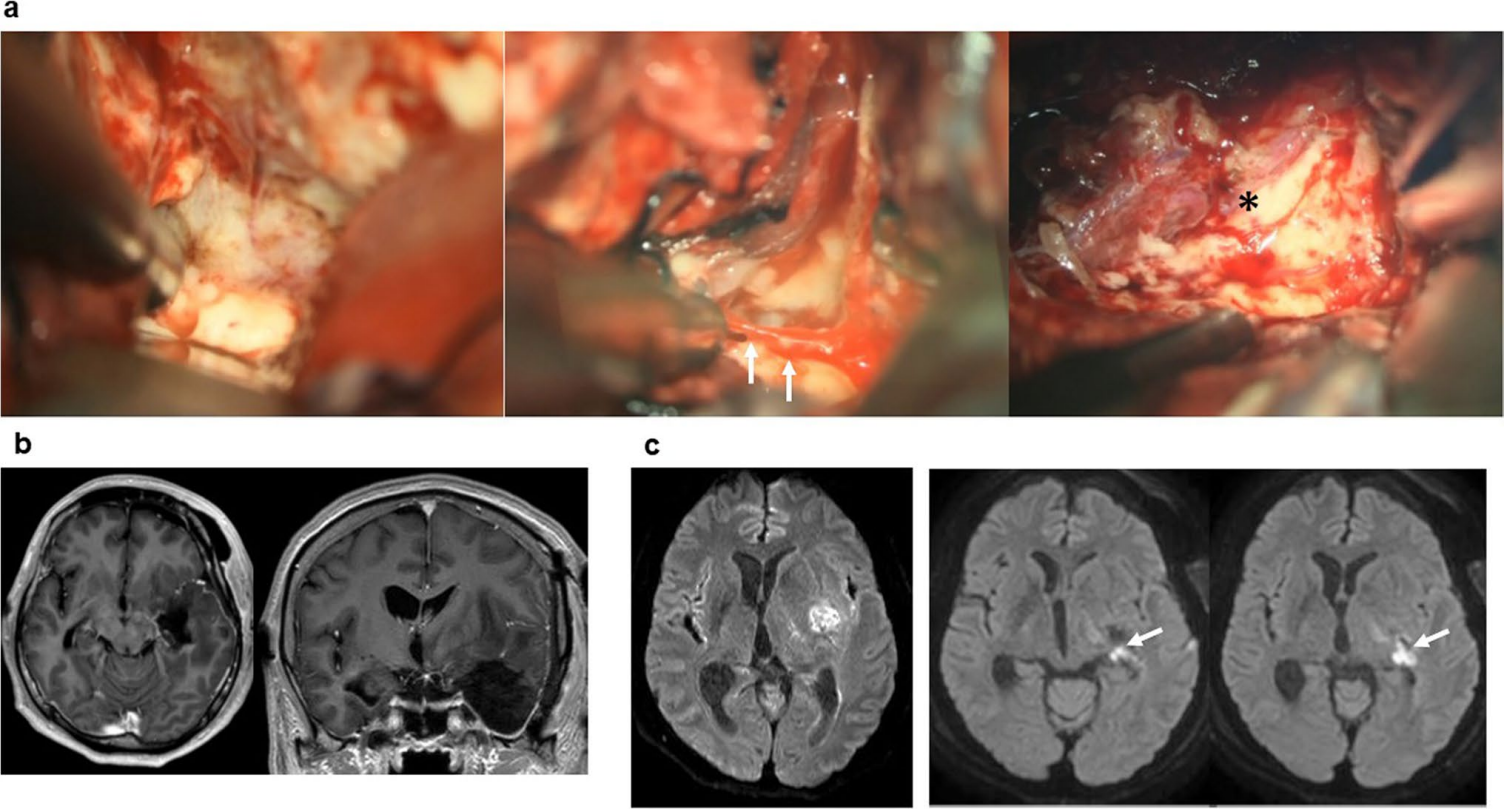
Intraoperative and perioperative magnetic resonance (MR) imaging findings. **a** Intraoperative views showing the distinct tumor border (left panel), feeding artery from the medial side (arrows, middle panel), and tumor location (right panel). The tumor was located anterocranial to the inferior horn of the left lateral ventricle (asterisk). **b** Postoperative gadolinium-enhanced T1-weighted MR images showing complete resection of the enhanced lesion. **c** Pre- (left panel) and postoperative (right panel) diffusion-weighted MR images (DWI) showing a new hyperintense lesion (arrows) in the retrolenticular and sublenticular internal capsule regions compared with preoperative DWI

DSA of the left ICA revealed tumor supply from a branch of the AchoA and localization anterior to the inferior choroidal point (Fig. 2a). MR angiography revealed a hypoplastic left posterior communicating artery (PcomA) and a well-developed left P1 segment supplying the posterior cerebral artery (PCA) (Fig. 2b). 3D-MIP demonstrated that the tumor was supplied solely by the AchoA, with no contribution from the ICA or middle cerebral artery (MCA) (Fig. 2c). The PT-LSAs and PT-AchoAs were clearly visualized on the 3D-MIP-PT images (Supplementary Video 1, Fig. 2c). Their spatial relationships to the tumor were further confirmed using sequential 2D fusion images (UHR-IA-CTA and 3D-Gd-T1WI; Supplementary Video 2) and 3D reconstructions combining UHR-IA-CTA, 3D-Gd-T1WI, and DTI (Fig. 2d). Both the PT-LSA and PT-AchoA were displaced anteriorly and medially, respectively, but were not encased by the tumor. Based on these findings, safe maximal resection was considered feasible with careful dissection along the tumor border. The patient underwent tumor resection via a left frontotemporal craniotomy under continuous motor evoked potential monitoring. Intraoperatively, the tumor was located anterior to the left inferior horn, had a well-defined border, and was supplied by medial arteries (Fig. 3a). Postoperatively, the patient developed right lower quadrant hemianopsia, while sensory aphasia gradually improved. MR image confirmed total resection of the enhancing lesion, with a small ischemic focus in the retrolenticular and sublenticular internal capsule (Fig. 3b).

Histopathological examination confirmed glioblastoma. The patient subsequently received concurrent radiotherapy and temozolomide, followed by maintenance temozolomide, and remained recurrence-free for 15 months.

## Discussion

In this case, we evaluated the anatomical relationship among the glioblastoma, its feeding arteries, and the PT-LSA and PT-AchoA using UHR-IA-CTA, Gd-T1WI, and DTI-based fiber tracking to determine the indication for surgical resection of an amygdala glioblastoma.

Our previous study demonstrated that long insular and medullary arteries—vessels thinner than the LSAs and the PT-AchoAs—can be successfully visualized using UHR-IA-CTA. Several alternative methods exist to visualize PAs via intra-arterial contrast injection. 3D-rotation angiography (3D-RA) using high-resolution angiography systems, such as Azurion 7 (Phillips, Amsterdam, Netherlands), offer improved imaging capabilities. Although 3D-RA lacks parenchymal detail, this limitation can be mitigated by fusing MR and 3D-RA images. With a spatial resolution of 150 μm in 2D imaging and 200 μm in 3D-RA, the comparative performance of these systems warrants consideration. To the best of our knowledge, no studies have directly compared the differences in the quality of imaging of PAs between high-resolution 3D-RA and UHR-IA-CTA. The maximum spatial resolution of both modalities is approximately 150 $\mu$m. However, because CT and angiography devices serve different purposes in clinical practice, their geometric properties and reconstruction methods differ. UHR-CT has smaller geometric properties (160 rows, 0.25 mm thickness: 40 mm) of the X-ray beam than 3D-RA, irrespective of the size of the flat panel detector. In addition, UHR-CT uses model-based iterative reconstruction [18] to reduce image noise and achieve superior image quality, including a higher signal-to-noise ratio (SNR), compared with conventional filtered back projection used in 3D-RA. Therefore, although invasive, we selected intra-arterial contrast injection combined with UHR-CT to achieve optimal SNR and detailed delineation of small perforating and medullary arteries. Because 3D-RA was not obtained in the present case, we demonstrated another representative case with an insular glioma. Following catheter placement in the left ICA, 3D-RA and UHR-IA-CTA were performed. Fusion images with tractography for PT and 3D-MIP images of LSAs were generated from both datasets, and the visualization of the LSAs was compared. The PT-LSAs were visualized more clearly using UHR-IA-CTA than using high-resolution 3D-RA, confirming its superior vessel delineation (Supplementary Fig. 1). Super-selective angiography can also provide detailed vascular mapping for surgical or endovascular planning. For example, selective injection of the anterior and posterior choroidal arteries allows differentiation between branches supplying normal brain tissue and those feeding arteriovenous malformations, facilitating safer embolization using the plexal point as an anatomical landmark and margin of safety [4]. However, this approach is limited by its small field of view and dependence on the skills of the examiner, which reduces its utility for preoperative mapping of vessels around glioblastoma. In contrast, UHR-IA-CTA offers wide-field visualization of the entire ICA territory, enabling comprehensive assessment of the vascular supply for PT, which receives blood supply from multiple vascular territories. Moreover, the automated intra-arterial injection used in UHR-IA-CTA ensures uniform vessel imaging with high reproducibility, minimizing variability associated with manual super-selective techniques. Thus, UHR-IA-CTA provides distinct advantages over 3D-RA and super-selective angiography in the preoperative vascular assessment of intraparenchymal tumors. From these differences, we believe that this approach is one of the most advanced and effective imaging techniques currently available for visualizing such fine vascular structures.

Determining the tumor’s precise location was challenging because of distortion of surrounding structures, including the inferior horn of the lateral ventricle, BG, and vessels in the ambient cistern. Accurate localization is crucial when planning resections of medial temporal tumors [13, 14]. Typically, tumors of the parahippocampal gyrus or hippocampus extend laterally, compressing but not invading the ventricle, whereas amygdala tumors tend to extend medially into the BG. UHR-IA-CTA provided objective visualization of the tumor’s position relative to the inferior choroidal point and its feeding arteries, aiding safe surgical planning.

A prior microanatomical study of 11 hemispheres reported that amygdala arteries originated from the MCA and AchoA in 100% of cases, from the terminal ICA in 64%, and from the P2 segment of the PCA in 9% [8]. Although the P2 segment of the PCA could not be evaluated in this case, the tumor was supplied by branches of the cisternal segment of the AchoA, without direct contribution from the ICA or MCA. Given the 150 µm maximal spatial resolution of UHR-CT and the mean diameter of amygdala arteries arising from the ICA and MCA (115–150 µm) [8], UHR-IA-CTA likely detected only the tumor-feeding arteries, while smaller amygdala branches may have remained undetectable because of their thin diameter. The AchoA supplied the medial and posterolateral regions of the amygdala in 82% of hemispheres, whereas MCA branches supplied the anterolateral region. This vascular configuration suggests that the tumor originated from the medial or posterolateral amygdala.

When a tumor is located in the amygdala, it may extend into the BG, anterior perforating substance, and the PT-LSAs and PT-AchoAs. Shibahara et al. reported that the resectability of medial temporal gliomas can be assessed by evaluating BG invasion [14]. In addition to direct evidence of BG involvement, they recommended assessing the tumor’s relationship to the basal vein or lateral ventricle. However, large tumors often obscure ventricular and venous landmarks. In this case, we distinguished the PT-AchoA from the tumor’s feeding arteries among multiple AchoA branches and identified the PT-LSAs, which were displaced anteriorly but not invaded. For glioblastoma resection, evaluating whether complete removal of the contrast-enhancing lesion is feasible is critical. When complete resection is possible, the tumor can be safely excised by dissecting along its macroscopic margins. Conversely, if arteries supplying the PT are involved, partial resection should be performed to preserve these vessels. Based on UHR-IA-CTA and intraoperative findings, we determined that complete resection was safe because the PA-PTs were not involved in the preoperative assessment and the tumor had a distinct border from normal brain on intraoperative findings.

This study has some limitations. First, the P2 segment of the PCA could not be assessed because of an adult-type PCA configuration. UHR-IA-CTA via the posterior circulation may be necessary to evaluate the tumor’s surrounding vasculature; however, simultaneous multi-vessel UHR-IA-CTA remains technically difficult due to residual contrast medium. Second, the patient developed postoperative ischemia in the retrolenticular and sublenticular internal capsule. The former is typically supplied by the lateral posterior choroidal artery or AchoA [3, 19, 20], whereas the latter is supplied by the AchoA. To clarify the mechanism of infarction, we examined the relationship between AchoA and ischemic lesions in the retrolenticular and sublenticular internal capsule. By overlaying the infarction site on preoperative UHR-IA-CTA images, we inferred that the ischemia likely resulted from intraoperative injury to small PAs from AchoA, based on the special relationship among the tumor, infarction, and AchoA (Supplementary Fig. 2). Third, the safety of UHR-IA-CTA has not yet been fully established. As this technique requires patient transfer from the angiography suite to the CT system, its risk profile may differ from that of conventional angiography, which is typically limited to the cervical arteries [15]. To establish its reliability to guide eligibility to surgery, prospective evaluation of its safety and clinical utility in larger patient cohorts is therefore warranted.

## Conclusion

The integration of UHR-IA-CTA, Gd-T1WI, and DTI-based tractography enables precise preoperative assessment of vascular anatomy in medial temporal glioblastoma. This multimodal approach supports surgical planning by clearly delineating the spatial relationships between the tumor and PT-PAs.

## Supplementary Information

Below is the link to the electronic supplementary material.

ESM 1 Supplementary Material 1 (DOCX 234 KB)

ESM 2 Supplementary Material 2: Rotational fusion of 3D maximum intensity projection vessel images from intra-arterial computed tomography angiography using ultrahigh-resolution computed tomography (UHR-IA-CTA) and pyramidal tract (PT) tractography. This video identifies tumor-feeding arteries arising from the AchoA (orange arrowheads), PT-supplying perforating artery from the AchoA (pink arrowhead), PT-supplying lenticulostriate artery (blue arrowhead), and posterior communicating artery (yellow arrowhead). (MP4 2.75 MB)

ESM 3 Supplementary Material 3: Sequential medial-to-lateral fusion images between intra-arterial computed tomography angiography using ultra-high-resolution computed tomography (UHR-IA-CTA; 2-mm-thick slabs) and gadolinium-enhanced 3D-T1-weighted magnetic resonance images. The red, pink, orange, blue, yellow, large white, and large green arrowheads and the red arrow indicate the anterior choroidal artery (AchoA), perforating artery of AchoA supplying the pyramidal tract (PT-AchoA), tumor-feeding arteries from the AchoA, PT-supplying lenticulostriate artery (PT-LSA), posterior communicating artery, left internal carotid artery, middle cerebral artery, and inferior choroidal point, respectively. The Gd-T1WI component is shown as a heat map. The PT-LSA was displaced anteriorly but not encased by the tumor, while the PT-AchoA ran along the medial tumor margin without evidence of encasement. (MP4 25.2 MB)

## Data Availability

No datasets were generated or analysed during the current study.
