# Supplementary material for: Preoperative assessment of perforating arteries around amygdala glioblastoma using intra-arterial CT angiography with ultra-high-resolution CT and MR tractography: a case report
Source: Acta Neurochir (Wien). 2025 Dec 11;167(1):320. doi: 10.1007/s00701-025-06741-y (PMC12700964; doi:10.1007/s00701-025-06741-y)
Supplement: Supplementary file 1 — Supplementary Material 1 (DOCX 234 KB) [file 701_2025_6741_MOESM1_ESM.docx]

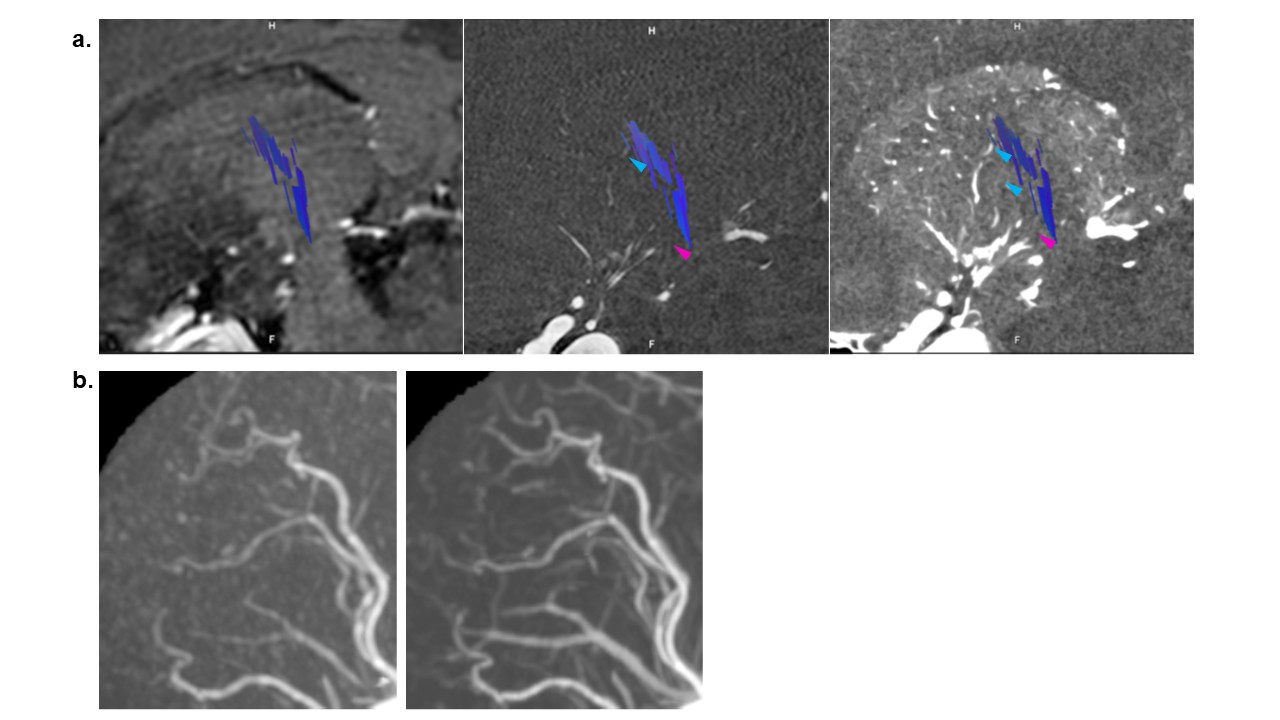
Supplementary Figure 1.

Comparison between 3D-rotation angiography (3D-RA) performed using a high-resolution angiography system (Azurion 7: Phillips) and intra-arterial computed tomography (CT) angiography using ultrahigh-resolution CT (UHR-IA-CTA) in another case of insular glioma. **a.** Fused sagittal images show T1-weighted magnetic resonance image (left panel), 3D-RA (middle panel), and UHR-IA-CTA (right panel) with pyramidal tract (PT) tractography. The lenticulostriate arteries (LSA) (blue arrowheads) and perforating arteries of the anterior choroidal artery (pink arrowheads) supplying the PT were more distinctly visualized on UHR-IA-CTA than on 3D-RA. **b.** Sagittal 3D-maximum intensity projection images of the anterior portion of LSAs from 3D--RA (left panel) and UHR-IA-CTA (right panel), demonstrating that the twigs of LSAs were visualized more clearly using UHR-IA-CTA than using high-resolution 3D-RA.


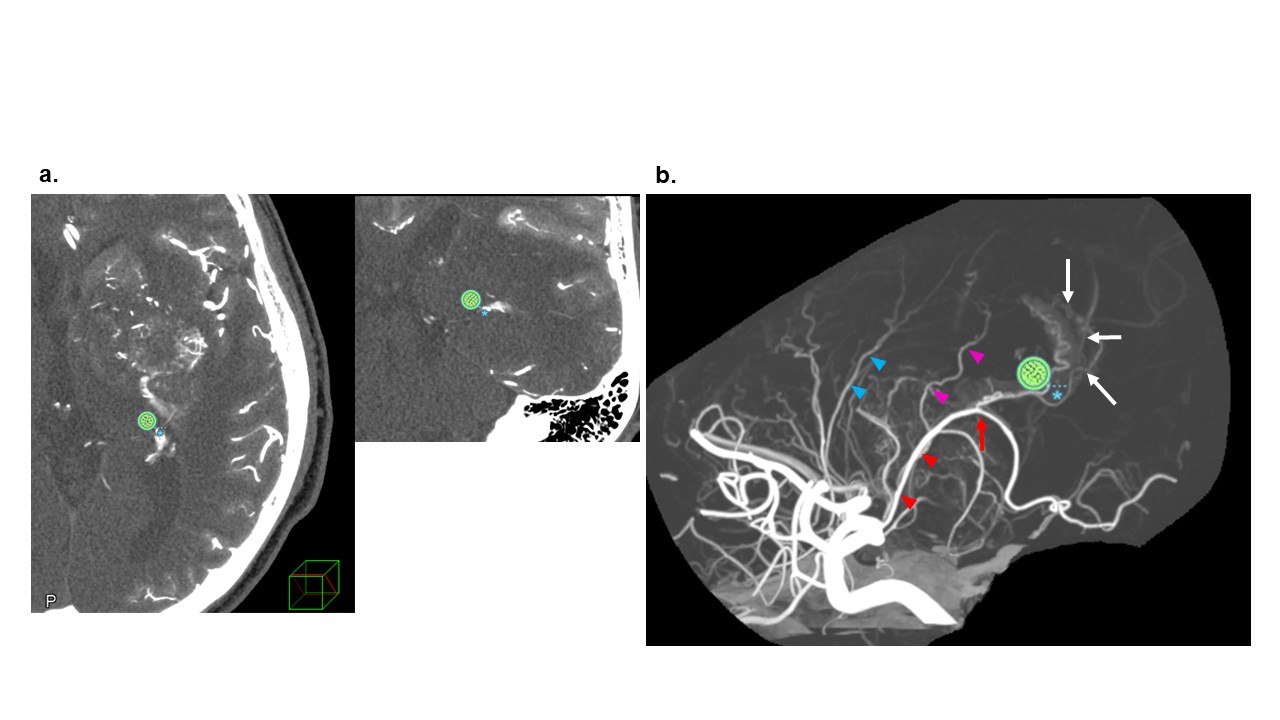


Supplementary Figure 2.

Anatomical relationship between postoperative infarction and the anterior choroidal artery (AchoA). **a.** Retrolenticular and sublenticular infarction after tumor resection (green circle) manually overlaid on preoperative intra-arterial computed tomography (CT) angiography using ultrahigh-resolution CT (UHR-IA-CTA). **b.** Sagittal maximum intensity projection images of arteries on UHR-IA-CTA with the overlaid infarction (green circle). Based on the spatial relationships among the tumor, infarction, and AchoA, the infarction likely resulted from intraoperative injury to a perforating artery arising from the AchoA during tumor resection. The blue, pink, red arrowhead, red arrow, and white arrows indicate the lenticulostriate artery, perforating artery of the AchoA supplying the pyramidal tract, AchoA, and choroid plexus, respectively.
